# Acetabular fractures in geriatric patients: epidemiology, pathomechanism, classification and treatment options

**DOI:** 10.1007/s00402-024-05312-7

**Published:** 2024-05-18

**Authors:** Dietmar Krappinger, Thomas Freude, Fabian Stuby, Richard A. Lindtner

**Affiliations:** 1 Department of Orthopaedics and Traumatology, Medical University of Innsbruck, Anichstraße 35, 6020 Innsbruck, Austria; 2 Department of Orthopaedics and Traumatology, Paracelsus Medical University Salzburg, Salzburg, Austria; 3 Department of Traumatology and General Surgery, BG Unfallklinik Murnau, Murnau Am Staffelsee, Germany

**Keywords:** Geriatric acetabular fracture, Anterior column and posterior hemitransverse fracture, Gull sign, Internal fixation, Primary arthroplasty

## Abstract

The incidence of geriatric acetabular fractures has shown a sharp increase in the last decades. The majority of patients are male, which is different to other osteoporotic fractures. The typical pathomechanism generally differs from acetabular fractures in young patients regarding both the direction and the amount of force transmission to the acetabulum via the femoral head. Geriatric fractures very frequently involve anterior structures of the acetabulum, while the posterior wall is less frequently involved. The anterior column and posterior hemitransverse (ACPHT) fracture is the most common fracture type. Superomedial dome impactions (gull sign) are a frequent feature in geriatric acetabular fractures as well. Treatment options include nonoperative treatment, internal fixation and arthoplasty. Nonoperative treatment includes rapid mobilisation and full weighbearing under analgesia and is advisable in non- or minimally displaced fractures without subluxation of the hip joint and without positive gull sign. Open reduction and internal fixation of geriatric acetabular fractures leads to good or excellent results, if anatomic reduction is achieved intraoperatively and loss of reduction does not occur postoperatively. Primary arthroplasty of geriatric acetabular fractures is a treatment option, which does not require anatomic reduction, allows for immediate postoperative full weightbearing and obviates several complications, which are associated with internal fixation. The major issue is the fixation of the acetabular cup in the fractured bone. Primary cups, reinforcement rings or a combination of arthroplasty and internal fixation may be applied depending on the acetabular fracture type.

## Epidemiology

The age distribution of patients sustaining acetabular fractures has dramatically changed in the last decades. From the 1960s to the 1990s, geriatric acetabular fractures were generally seen as rare injuries with young male patients representing the predominant patient group [1, 2]. This period was followed by a period with a sharp increase of the incidence of geriatric acetabular fractures starting in the 1990s [3–13]. In the first decade of the recent century it was estimated by several authors that elderly patients will soon be the typical age group of patients sustaining acetabular fractures [3, 6, 8, 14].

This prediction has become true in the meantime [11, 15]. Sullivan found that geriatric acetabular fractures increased by 67% between 1996 and 2014 [8]. Using data from the German Pelvic Registry, it was found that more than 50% of all acetabular fracture patients were older than 60 years and that the age peak was found at 75–80 years [11]. Applying data from the same registry, Stöckle found that the median age of patients sustaining acetabular fractures was 49 years in 2004 and 77 years in 2017 [16]. Accordingly, the median age increased by 28 years within 13 years only. General demographic developments towards an older population as well as a medical progess with better survival rates following the first hip fracture account for these findings [17, 18].

Accordingly, the recent age distribution of patients sustaining acetabular fractures shows a typical bimodal distribution. Young patients suffer from acetabular fractures following high-energy trauma, while the elderly suffer from acetabular fractures following low-energy trauma [19, 20]. A shift of the age distribution of fracture patients towards the elderly is typically accompanied with a shift of the gender distribution towards female patients. This is, however, not true for geriatric acetabular fracture patients. Ochs found a constant gender ratio in the course of time with a vast majority of geriatric acetabular fracture patients being male (78% vs 22%) [2]. This finding was confirmed in recent studies [15, 21]. A plausible explanation may be the worse mean bone quality in female geriatric patients, which results in proximal femoral fractures after simple falls, while geriatric male patients more frequently suffer from acetabular fractures instead.

### Pathomechanism

The typical pathomechanism of geriatric acetabular fractures generally differs from acetabular fractures in young patients regarding both the direction and the amount of force transmission to the acetabulum via the femoral head.

Osteoporosis is ubiquitous in geriatric patients. Hence, the vast majority of geriatric acetabular fractures are osteoporotic fractures following low-energy trauma [4, 7, 22, 23]. The typical injury mechanism involves a simple fall from standing height with the hip joint in extension and neutral rotation [13–15, 23]. This injury mechanism results in force transmission via the greater trochanter to the femoral neck and femoral head [4, 9, 19, 24]. Considerung the femoral neck anteversion and the neck-shaft angle, the primary force vector is directed to the anterior and superior part of the acetabulum [25].

Accordingly, geriatric fractures very frequently involve anterior structures of the acetabulum (i.e. anterior column, anterior wall and medial wall), while the posterior wall is less frequently involved [2–4, 6, 9, 13, 15, 19, 24, 25]. The typical fracture type in geriatric acetabular fractures is the anterior column and posterior hemitransverse (ACPHT) fracture with intrapelvic dislocation of the femoral head, which is described in detail in the next section.

Associated with a decrease in bone quality is also an increase in the rate of comminution and articular impactions in geriatric acetabular fractures [3, 4, 6, 7, 9, 14, 15]. This includes both the acetabulum and the femoral head. These fracture characteristics influence the feasibility of an anatomical reduction of the fracture and the outcome after internal fixation and is therefore a relevant parameter in the decision-making [17]. The typical type of articular impaction in geriatric acetabular fractures is the superomedial dome impaction, which is known as „gull sign “. It is discussed in the following sections.

### Fracture types and classification

It was shown that classification systems, which were developed for pelvic ring injuries after high-energy trauma, for example the AO/OTA classification and the Young and Burgess classification, are not suitable for the classification of osteoporotic pelvic ring fractures [26, 27]. Accordingly, classification systems were specifically developed for the assessment of geriatric pelvic ring fractures and are now widely used [28, 29]. The standard classification system for acetabular fractures is the Judet and Letournel classification. It was shown that this classification system reliably works for the classification of geriatric acetabular fractures as well. Accordingly, there is no need to develop and learn new classification systems for acetabular fractures in the elderly. The relative frequency of fracture types within the Judet and Letournel classification, however, differ between young and geriatric acetabular fracture patients. The involvement rate of the anterior structures of the acetabulum is higher in geriatric patients, as described in the previous section [9, 26].

**Fig. 1:**
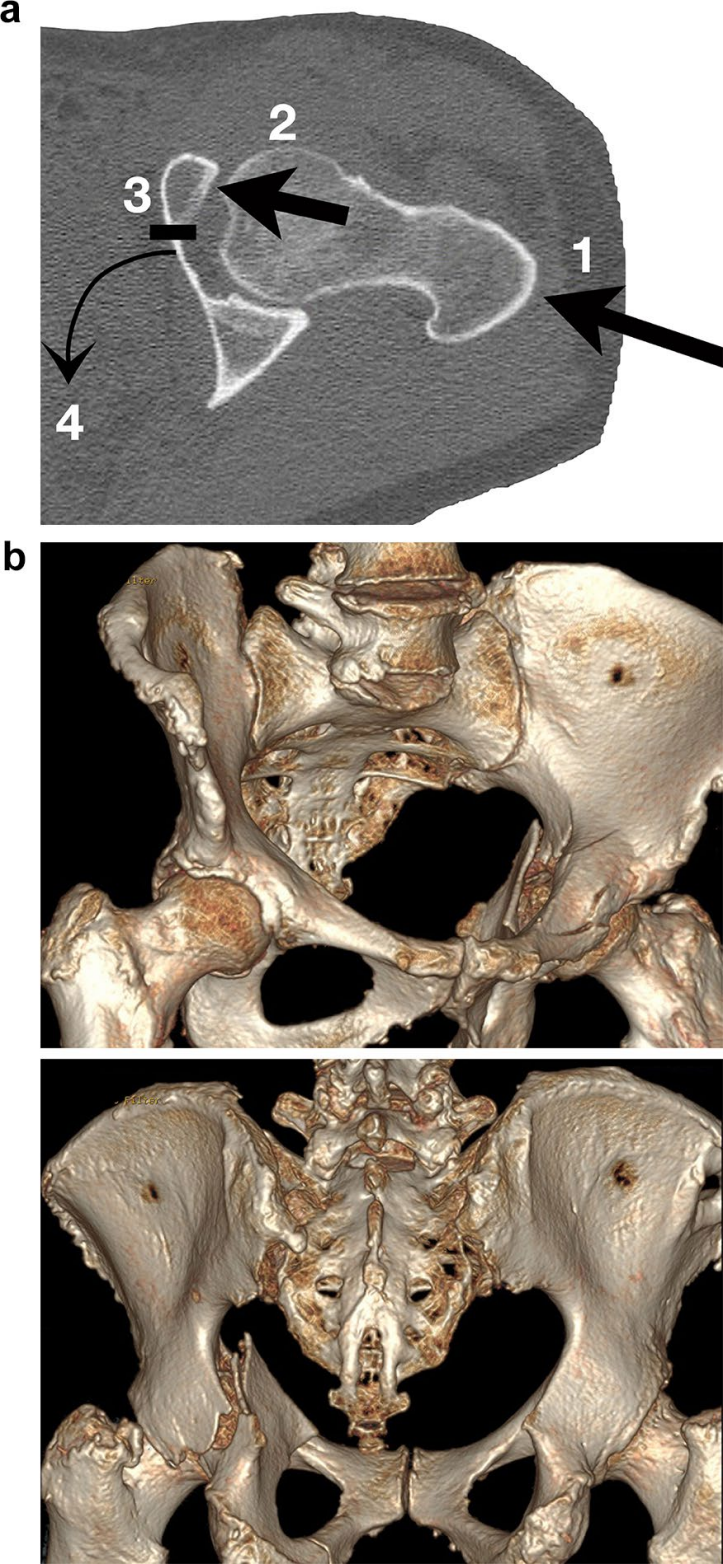
**a** Injury mechanism of ACPHT fractures: (1) Force transmission via the greater trochanter. (2) Direction of the force vector to the anterior and superior part of the acetabulum. (3) Fracture between the anterior column and the medial wall. (4) Posterior hemitransverse fracture and internal rotation of the posterior column. **b** Typical fracture characteristics of ACPHT fractures: multifragmentary anterior column fracture, superomedial articular impaction (“gull sign”), medial wall and posterior column in osseous continuity, simple posterior hemitransverse fracture, internal rotation of the posterior column and the medial wall

**Fig. 2:**
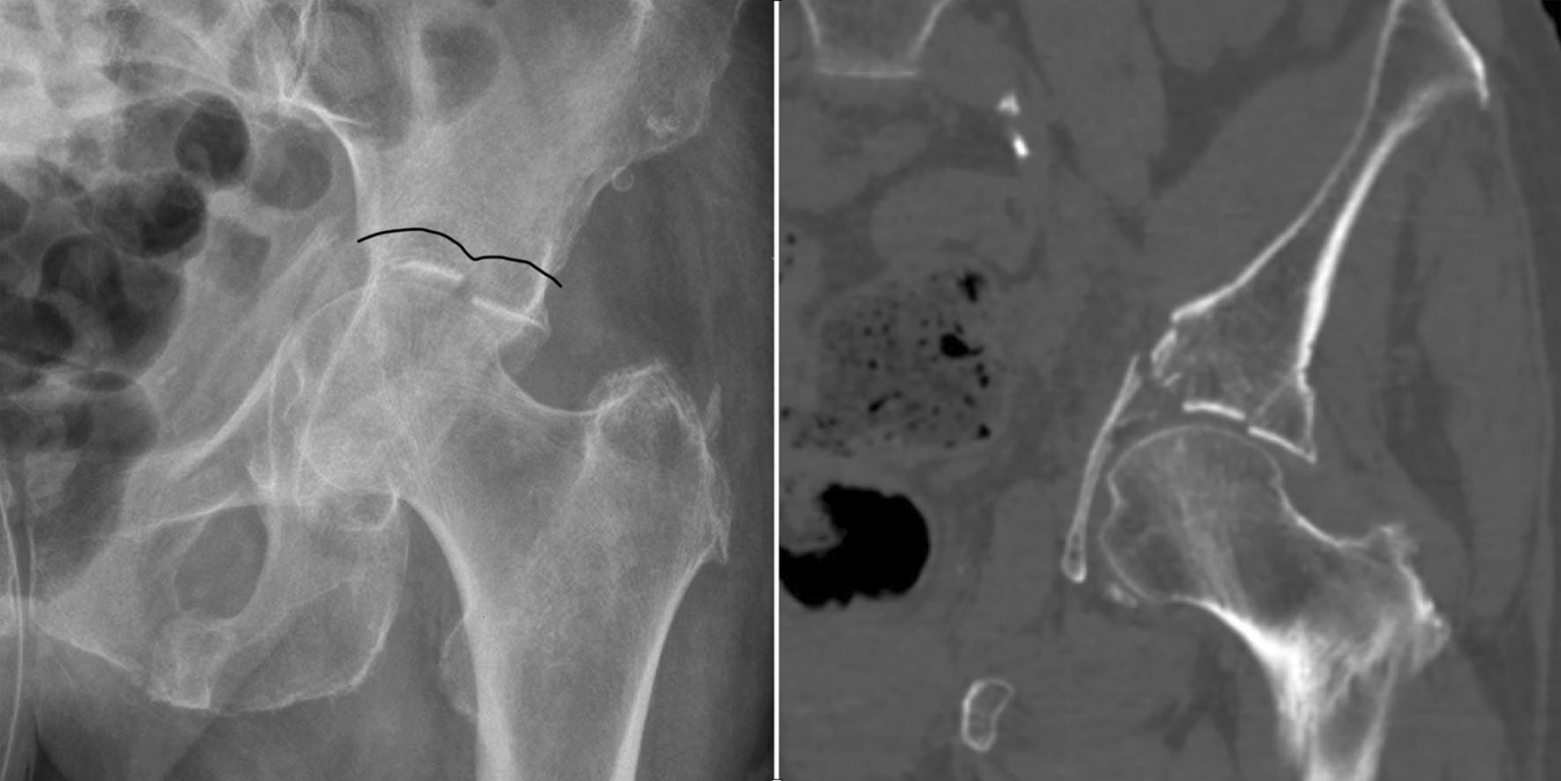
Superomedial dome impactions aka “gull sign”. The name is derived from the typical schematic drawing of gulls by children

**Fig. 3:**
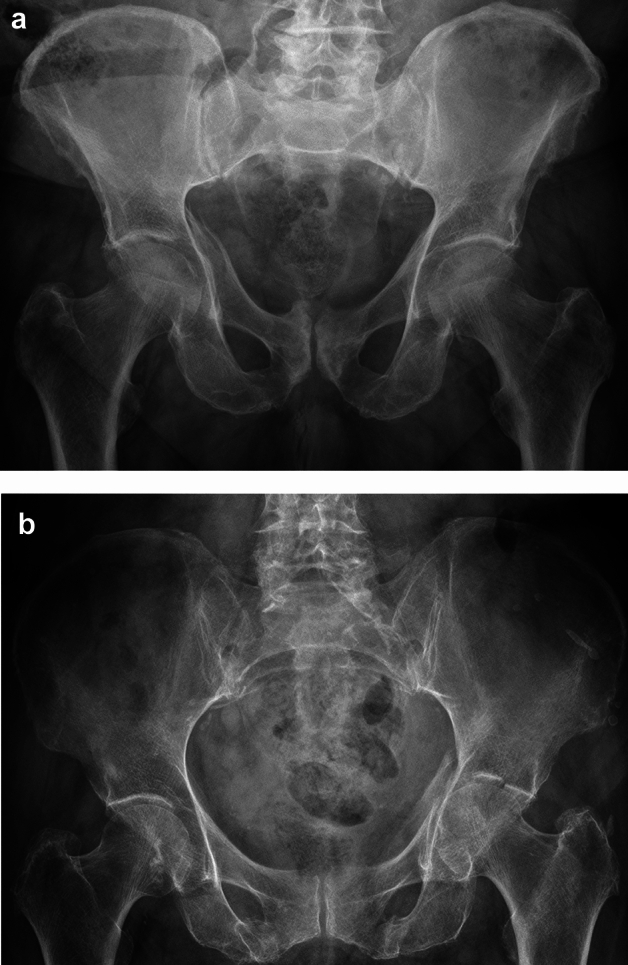
**a** Minimally displaced ACPHT fracture without gull sign. The fracture healed without further displacement after nonoperative treatment. **b** Minimally displaced ACPHT fracture with gull sign. Nonoperative treatment resulted in massive pain and further subluxation. Primary arthroplasty was performed after eight days

**Fig. 4:**
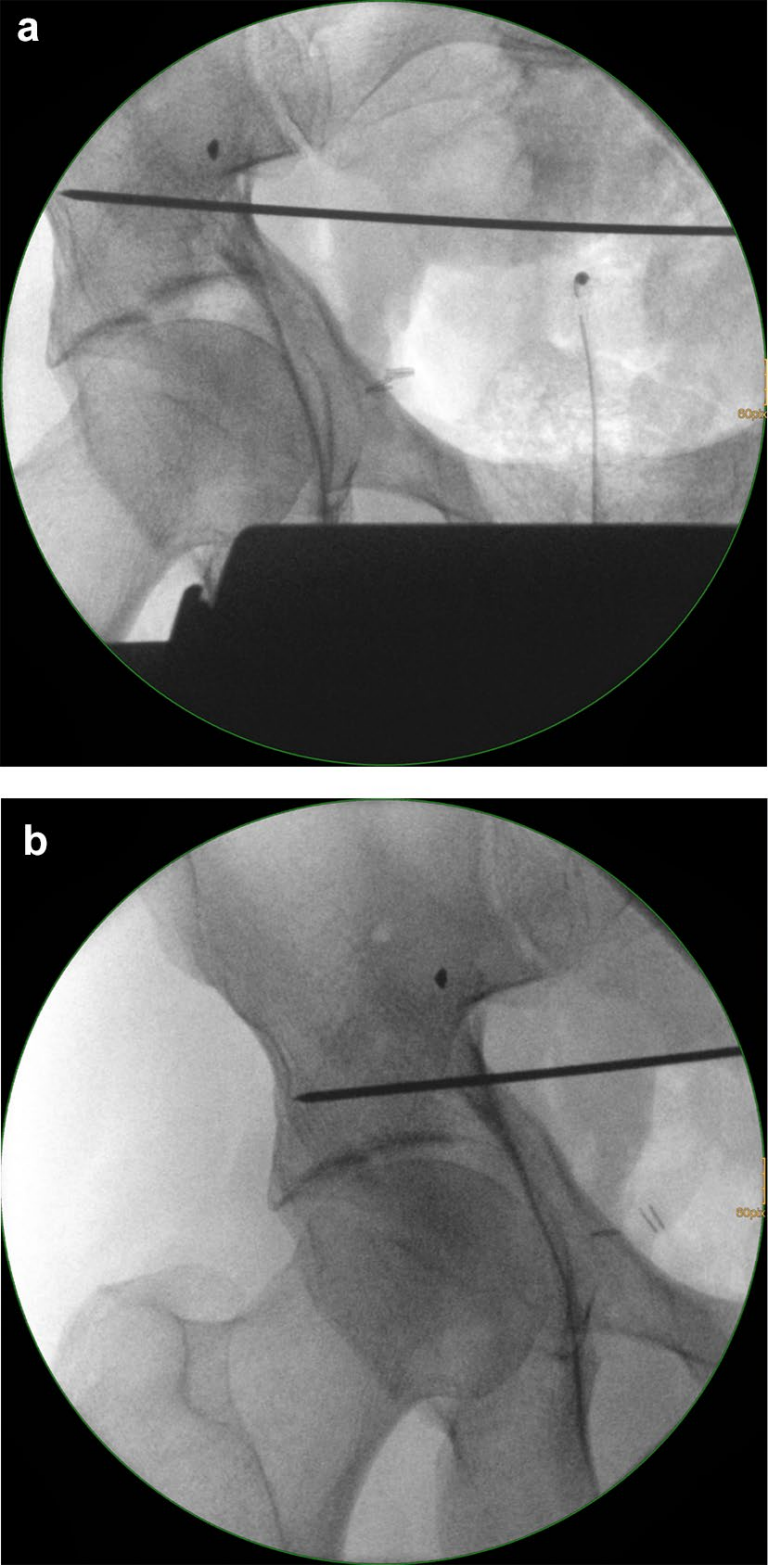
**a** Unreduced superomedial dome impaction after reduction of the anterior column and temporary wire fixation. **b** Improved reduction of the superomedial dome impaction, but with a remaining moderate gull sign and a high risk of loss of reduction in geriatric patients

The most frequent acetabular fracture type in geriatric patients is the anterior column and posterior hemitransverse (ACPHT) fracture [15, 17, 24, 25, 30–34]. Despite its cumbersome name, ACPHT follows a uniform injury mechanism. Following simple falls to the lateral side, force transmission starts at the greater trochanter (Fig. 1a, b). It follows the femoral neck and the femoral head [4, 9, 19, 24]. With the leg in neutral rotation and considering the femoral neck anteversion and the neck-shaft angle, the primary force vector is directed to the anterior and superior part of the acetabulum (Figs. 1a, 2). This frequently results in multifragmentary anterior column fractures and articular impactions of the superomedial dome (“gull sign”). Onward force transmission leads to a fracture between the anterior column and the medial wall (Figs. 1a, 3). Additionally, the eponymous hemitransverse fracture of the posterior column allows for an internal rotation of the posterior column (Figs. 1a, 4). Accordingly, the medial wall is not “medialized” or “centralized” in its entirety following a translational displacement, as stated by several authors. Instead, the medial wall is in osseous continuity with the posterior column and shows a rotational displacement due to the internal rotation of the posterior column. Fig. 1b shows a 3D reconstruction of a typical ACPHT with the following fracture characteristics:

- Multifragmentary anterior column fracture,
- Superomedial articular impaction (“gull sign”),
- Medial wall and posterior column in osseous continuity,
- Simple posterior hemitransverse fracture,
- Internal rotation of the posterior column and the medial wall.

A superomedial dome impaction was first named as “ gull sign ” by Anglen in 2003 [4, 33, 35]. It is a frequent feature in geriatric acetabular fractures, but occurs in high-energy acetabular fractures in young patients as well. The name is derived from the typical schematic drawing of gulls by children (Fig. 2). The presence of a gull sign has several implications for the decision-making in geriatric acetabular fracture patients as discussed in the following sections:

- It is a sign of articular subluxation (Section “Nonoperative treatment”),
- It is a risk factor for failure of internal fixation (Section “Internal fixation”),
- The superolaterale dome is in osseous continuity with the supraacetabular bone (Section “Arthroplasty”).

## Treatment options

The following treatment options for geriatric acetabular fractures are available in general:

- Nonoperative treatment,
- Internal fixation,
- Arthroplasty.

Accordingly, the decision for either nonoperative or operative treatment needs to be made first. After deciding for surgical treatment, the decision has to be made for either internal fixation, arthroplasty or a combination of both.

### Nonoperative treatment

Nonoperative treatment was the sole treatment option for geriatric acetabular fractures for decades. Judet and Letournel, for example, recommended in the 1960s the avoidance of any surgery in osteoporotic acetabular fractures [36]. They partially revised their opinion in the second edition of their well-known text book in 1993 by stating that age alone should not be seen as a contraindication for acetabular fracture surgery. „Osteopenia of the innominate bone “, however, was still seen as a contraindication.

Non- or minimally displaced acetabular fractures without subluxation of the hip joint are still treated nonoperatively with pain therapy and rapid mobilisation in order to prevent secondary complications due to immobilisation [1, 3, 4, 14, 15, 19, 21, 22, 32, 33, 37, 38]. Full weightbearing should be allowed in geriatric patients, as these patients are generally not able to perform partial weightbearing [39, 40]. Satisfactory to good or even excellent outcome can be expected in these cases [21, 32, 33].

Displaced geriatric acetabular fractures were typically treated with skeletal traction and prolonged bed rest for 6–12 weeks. The functional results, however, were poor in the majority of cases [41] mainly due to the following two reasons. First, adequate reduction was not achieved by closed reduction and traction [3, 4, 22, 42]. Acetabular fractures mainly show rotational and not translational deformities, as decribed in the previous section. Accordingly, traction is not very effective in achieving adequate reduction of displaced acetabular fractures [5, 14, 19, 32]. Exceptional cases may be associated both-column fractures, which may show secondary congruence of the articular surface after closed reduction [3, 4, 6, 13, 19, 32, 34]. Second, prolonged bed rest and traction is associated with a high risk of secondary complications due to immobilisation [1, 4, 5, 12, 19, 22, 32, 34, 37, 42]. These complications include pneumonia, urinary tract infection, bed sores, DVT, sarcopenia and a general loss of the ambulatory function and an increase in social dependency. They are associated with a 1-year mortality rate of 20–40% after nonoperative treatment of geriatric acetabular fractures [43]. Accordingly, bed rest and traction for weeks should not be advised in a modern ortho-geriatric treatment concept.

Fractures with a positive gull sign have to be classified as displaced fractures with subluxation of the hip joint in this regard. The femoral head follows the impacted superomedial dome (the medial wing of the gull). The uninjured superolateral dome (the lateral wing of the gull) is intact and in osseous continuity with the supracetabular bone and the iliac wing. The femoral head is subluxated related to the superolateral dome. Accordingly, nonoperative treatment of geriatric acetabular fractures with positive gull signs ist not advisable (Fig. 3).

In summary, nonoperative treatment of geriatric acetabular fractures, i.e. rapid mobilisation and full weighbearing under analgesia, is advisable in non- or minimally displaced fractures without subluxation of the hip joint and without positive gull sign. Additionally, it may be suitable for patients, who were already bedridden prior to the acetabular trauma. Surgery, i.e. internal fixation, arthroplasty or a combination of both, should be considered in all other cases.

### Internal fixation

Open reduction and internal fixation is the treatment of choice for displaced acetabular fractures in nongeriatric patients. It allows for an anatomic reduction of the fracture and early postoperative mobilisation. Additionally, the acetabulum has—in contrast to displaced femoral neck fractures—a very good osseous healing potential with a low nonunion rate [6, 42]. The general principles of internal fixation in nongeriatric patients apply for geriatric patients as well. Anterior approaches, however, are more frequently used in the elderly considering the typical fracture types in this patients group. Buttressing of the medial wall is the most important measure in order to prevent postoperative loss of reduction [7, 15, 19, 25, 30]. The anterior intrapelvic approach (widely known as Stoppa approach) is therefore advisable due to the exposure of the medial wall provided by this approach [7, 25]. Buttressing of the medial wall may be either achieved by using periarticular infracetabular screws [30], quadrilateral plates [15, 19] or posterior column screws [44]. Angular stable precontoured plates may additionally facilitate internal fixation of osteoporotic acetabular fractures in the future [19].

The clinical outcome following open reduction and internal fixation of acetabular fratures mainly depends on the following parameters:

- Fracture-related parameters,
- Surgery-related parameters,
- Patient-related parameters.

Fracture-related parameters associated with a poor outcome are (1) hip dislocation, (2) posterior wall fractures, (3) marginal impactions, (4) femoral head impactions and (5) a positive gull sign [3–7, 14, 19, 21, 23, 33, 35]. The most important surgery-related parameter for a beneficial outcome is anatomic reduction of the fracture [2–4, 19, 32, 45, 46]. Especially superomedial dome impactions (gull sign) pose a massive challenge for the surgeon to achieve anatomic reduction without residual subluxation of the femoral head and without subsequent loss of reduction during postoperative mobilization (Fig. 4) [7, 35, 47, 48]. Anglen, for example, found that a positive gull sign was predictive for failure of reduction and fixation in all cases in acetabular fracture patients aged > 65 years [35]. Patient-related parameters with adverse effects on the functional outcome include osteoporosis, relevant co-morbidities, impaired cognitive function, preexisting osteoarthritis and the inability to perform postoperative partial weightbearing [1, 4, 6, 23, 42].

Open reduction and internal fixation of geriatric acetabular fractures leads to good or excellent results, if anatomic reduction is achieved intraoperatively and loss of reduction does not occur postoperatively [3, 4, 14, 15, 19, 23, 33, 42]. Unfortunately, the abovementioned risk factors for adverse events are very common in this patients' collective. Especially anatomic reduction is less frequently achieved in the elderly [2–4, 33, 40, 45, 47]. Ochs, for example, found a significant correlation between increasing age and decreasing quality of reduction [2].

It is therefore not surprising that the functional outcomes after internal fixation of acetabular fractures are much worse in the elderly compared to young patients [4, 6, 17, 18, 23, 40, 46, 49]. Accordingly, there is a high rate of conversion to arthroplasty after internal fixation of acetabular fractures (secondary arthroplasty), if anatomic reduction was not achieved. The rates range from 23 to 54% in the literature [6, 9, 19, 46, 48].

Secondary arthroplasty may be a viable treatment concept in young patients. Internal fixation and osseous healing of the fracture without gross deformity of the acetabulum provides a good bone stock for the fixation of a primary cup. In geriatric patients, however, a two-staged treatment concept is not advisable [6]. The concept of “single-shot surgery”, i.e. a single surgical intervention as soon as possible followed by immediate postoperative full weightbearing, should be preferred in the elderly. The concept of primary arthroplasty in geriatric femoral neck fractures, for example, applies this concept. The concept of primary arthroplasty in geriatric acetabular fractures follows this concept as well.

### Arthroplasty

Primary arthroplasty of geriatric acetabular fractures is a treatment option, which does not require anatomic reduction and allows for immediate postoperative full weightbearing [1, 4, 14, 18]. It is indicated, if anatomic reduction may not be achievable intraoperatively or loss of reduction may not be avoidable postoperatively due to multifragmentary and comminuted fracture patterns or due to articular impactions [3, 18]. This may be true for the vast majority of displaced acetabular fractures in the elderly according to the author´s opinion. This topic, however, is controversially discussed in the literature with high-qualtity studies still missing.

Primary arthroplasty of acetabular fractures obviates several complications, which are associated with internal fixation [5, 14, 17]. These complications mainly involve non-anatomic reduction, postoperative loss of reduction and the development of posttraumatic osteoarthritis. Primary arthroplasty of acetabular fractures, however, is associated with its own distinct complications. The major issue is the fixation of the acetabular cup in the fractured bone with consecutive loosening of the cup [4, 14, 17, 19, 22, 23, 47].

In general, there are three options for the fixation of the acetabular cup:

- To apply a primary uncemented or cemented cup,
- To use a reinforcement ring,
- To combine internal fixation and arthroplasty.

It depends on the acetabular fracture type, which mode of acetabular cup fixation is suitable [19]. Surpisingly, there are no studies in the literature addressing acetabular cup fixation depending on fracture types. Primary arthroplasty of the following types of geriatric acetabular fractures will be discussed:

- Posterior wall fracture,
- Anterior column and posterior hemitransverse fracture (ACPHT),
- Both-column fracture.

**Fig. 5:**
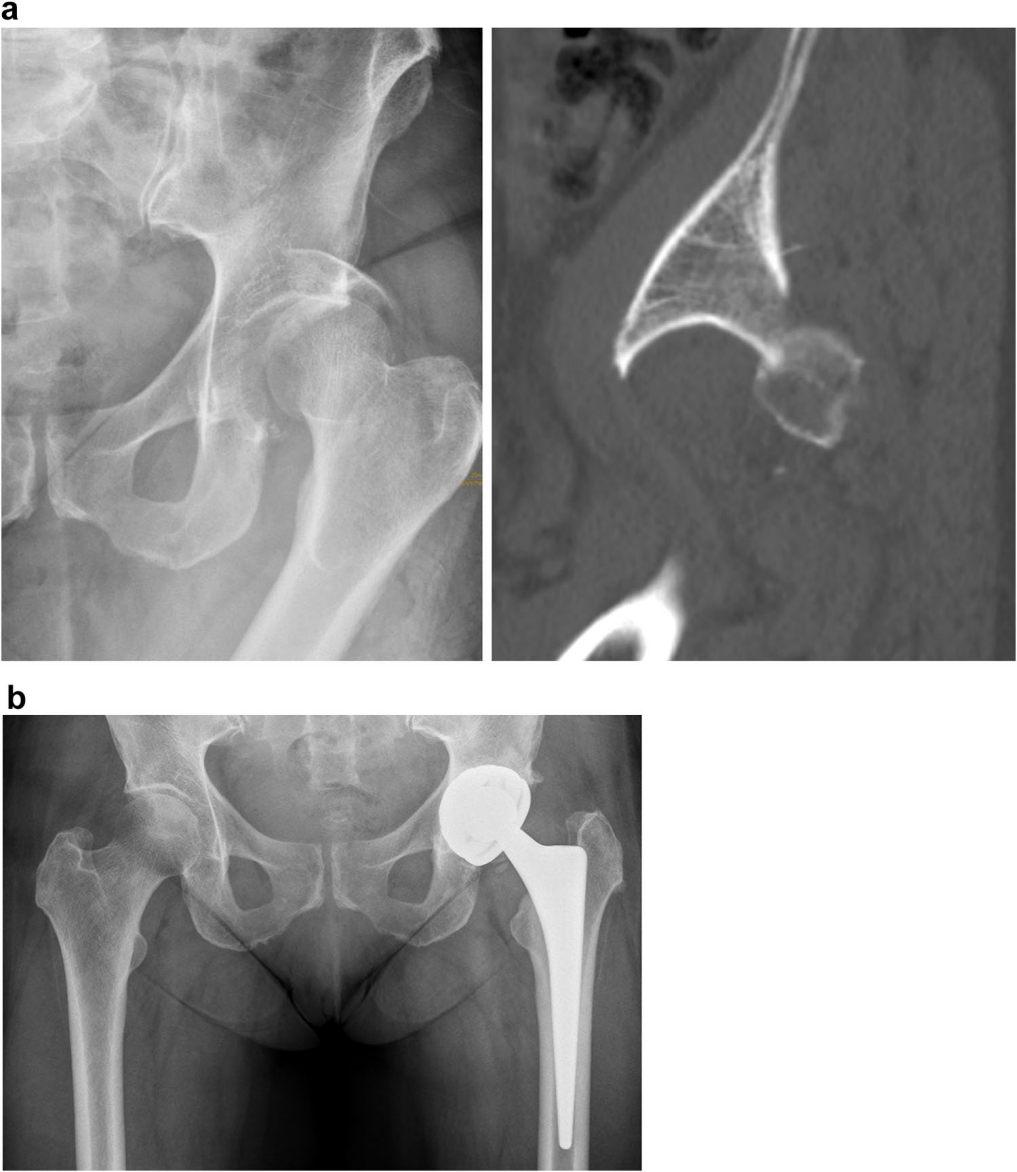
**a** Posterior hip dislocation with posterior wall fracture and femoral head impaction in an 86 year old female. **b** Primary arthoplasty without reinforcement rings or additional internal fixation using a double mobility system

Posterior wall fractures are not very common in geriatric patients, but they are not absolutely unique neither. The typical injury mechanism is a fall downstairs. Simple posterior wall fractures obviously allow an anatomic reduction via a posterior approach. These injuries, however, have a very high rate of femoral head impactions with a high rate of posttraumatic osteoarthritis (Fig. 5a) [3, 5, 6, 50]. Additionally, primary arthroplasty of posterior wall fractures does neither require reinforcement rings nor an additional internal fixation. The use of double mobility systems is advisable in order to prevent postoperative posterior hip dislocation (Fig. 5b).

**Fig. 6:**
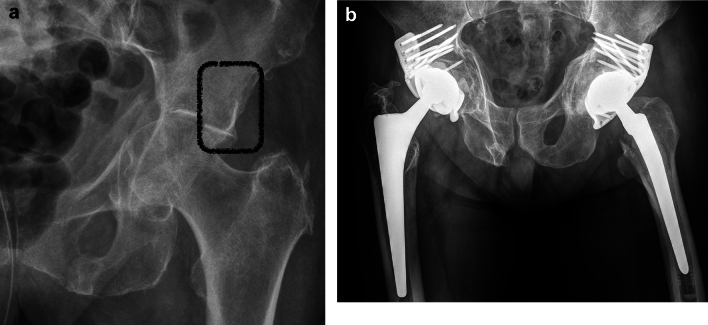
**a** ACPHT fracture with gull sign. The superolateral dome is intact and in osseous continuity with the supracetabular bone and the iliac wing. **b** Primary arthoplasty of ACPHT fractures using angular stable reinforcement rings. X-ray control after five years (right) and one year (left)

ACPHT fractures represent the typical displaced geriatric acetabular fracture type. Primary arthroplasty is often advisable due to multifragmentary fracture patterns and the frequent occurrence of a positive gull sign. The fracture characteristics of ACPHT fractures with positive gull sign are shown in Figs. 1, 2. While the superomedial dome (the medial wing of the gull) is impacted, the superolateral dome (the lateral wing of the gull) is intact and in osseous continuity with the supracetabular bone and the iliac wing (Fig. 6a). This allows a reinforcement ring to be fixed in this bony area without additional internal fixation. In the first author´s institution, an angular stable reinforcement ring specificially designed for osteoporotic acetabular fractures with multiple screws is used (ARRP, 41Medical, Bettlach, Switzerland, Fig. 6b). The ring acts as an internal fixation device and allows bone healing without formally reducing the fracture [51, 52]. The same principle applies for transverse fractures, which typically occur as periprosthetic fractures, as well.

**Fig. 7:**
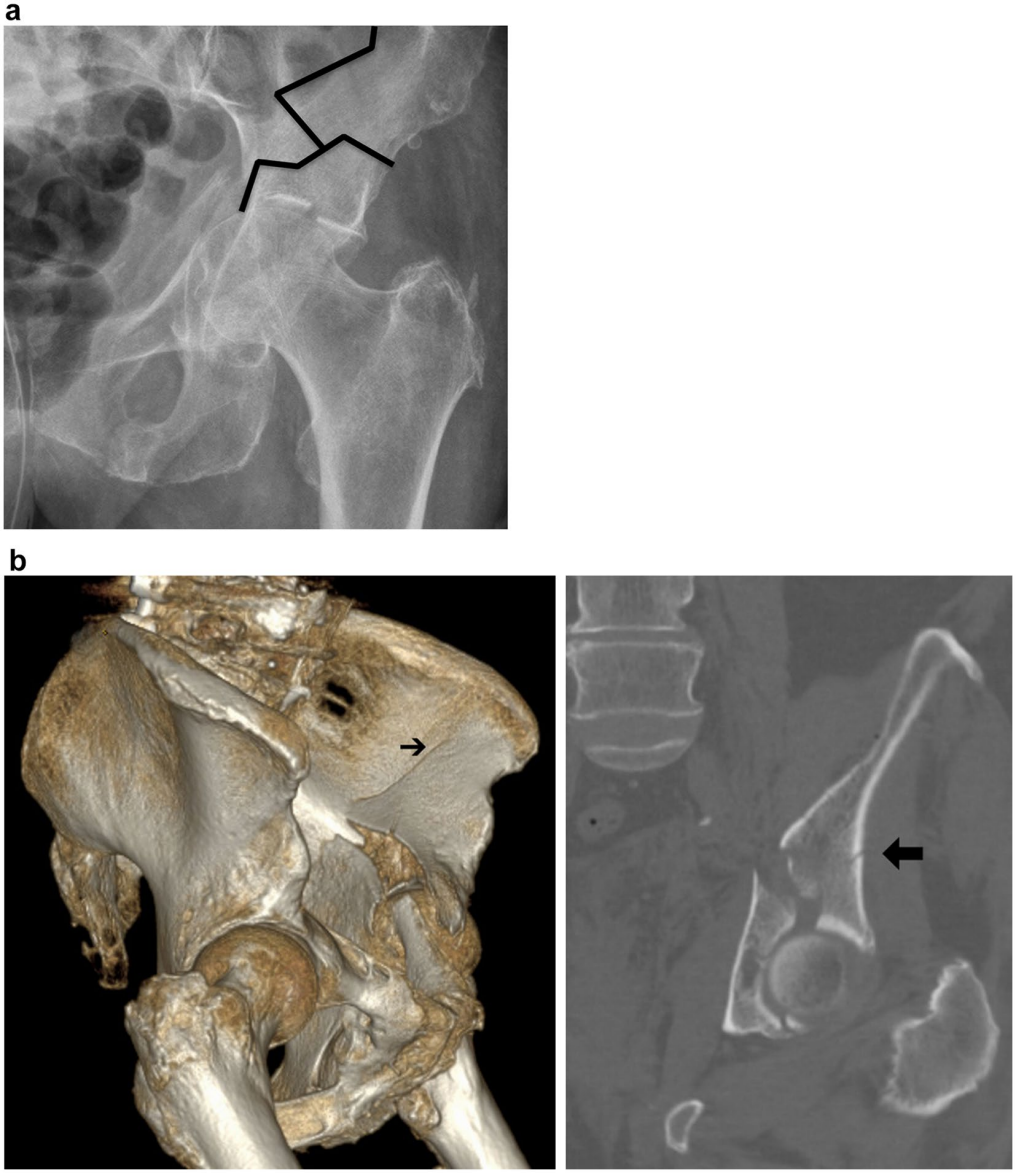
**a** Both-column fractures are defined as fractures with a bony separation of the entire acetabulum from the remaining iliac bone. **b** In geriatric both-colum fractures the supracetabular fracture components are frequently incomplete and undisplaced and therefore inherently stable

Both-column fractures are defined as fractures with a separation of both columns from each other in combination with a separation of the entire acetabulum from the remaining iliac bone. Accordingly, there is—in contrast to ACPHT fractures—no osseous continuity of the acetabulum with the supracetabular bone and the iliac wing (Fig. 7a). Therefore, the use of reinforcement rings without additional internal fixation is generally not advisable in both-column fractures. In geriatric patients, however, both-column fractures frequently follow the injury mechanisms of ACPHT fractures (Figs. 1, 2) with additional nondisplaced and incomplete proximal fracture components (Fig. 7b). These fracture components are inherently stable and allow for the use of reinforcement rings without further internal fixation according to the first author´s experience. If the supraacetabular fracture components are displaced and unstable, additional internal fixation is mandatory.
